# Continual Deletion of Spinal Microglia Reforms Astrocyte Scar Favoring Axonal Regeneration

**DOI:** 10.3389/fphar.2022.881195

**Published:** 2022-06-27

**Authors:** Longkuo Xia, Jianhuan Qi, Mingming Tang, Jing Liu, Da Zhang, Yanbing Zhu, Baoyang Hu

**Affiliations:** ^1^ State Key Laboratory of Stem Cell and Reproductive Biology, Institute of Zoology, Chinese Academy of Sciences (CAS), Beijing, China; ^2^ Institute for Stem Cell and Regeneration, Chinese Academy of Sciences, Beijing, China; ^3^ University of Chinese Academy of Sciences, Beijing, China; ^4^ Beijing Institute for Stem Cell and Regenerative Medicine, Beijing, China; ^5^ Beijing Clinical Research Institute, Beijing Friendship Hospital, Capital Medical University, Beijing, China; ^6^ National Stem Cell Resource Center, Institute of Zoology (CAS), Beijing, China

**Keywords:** astrocyte scar, microglia, spinal cord injury, scar-formed astrocyte, collagen I

## Abstract

Astrocyte scar formation after spinal cord injury (SCI) efficiently limits the accurate damage but physically restricts the following axon regeneration. Lately, fine tuning scar formation is becoming a novel strategy to develop SCI treatment, yet how to leverage these opposite effects remains challenging. Here, utilizing an improved drug administration approach, we show that in a mouse model of spinal cord injury, continual deletion of microglia, especially upon scar formation, by pexidartinib decreases the amount of microglia-derived collagen I and reforms the astrocyte scar. The astrocytes become less compacted in the scar, which permits axon regeneration and extension. Although continual microglia deletion did not significantly improve the locomotive performance of the SCI mice, it did ameliorate their weight loss, possibly by improving their relevant health conditions. We thus identified a novel approach to regulate astrocyte scars for improved axon regeneration, which is indicative of the clinical treatment of SCI patients.

## Introduction

Spinal cord injury (SCI) results in physical disability and impaired functions of various organ systems. Glial cells in the damaged area are activated violently, of which the activated astrocytes proliferate and become hyperactive, enter scar-forming states, and strongly bond with each other to form a dense structure that seals the damaged areas and prevents damage-associated molecular spreading to the surrounding intact tissues (Silver and Miller, 2004; Hara et al., 2017; Gaudet and Fonken, 2018). This structure, termed astrocyte scar, limits the spread of damaged areas but also shuts off the transmission of upstream and downstream signals of the spinal cord tract, resulting in sensory and motor dysfunction below the injury segment level (Escartin et al., 2021). In addition, glial scars block the regeneration of axons to the injured area during post-injury recovery, resulting in incomplete structural and functional reconstruction of the injured center area (Silver and Miller, 2004). Therefore, research on astrocyte scars has carried the hope of healing SCI.

Currently, it is difficult to balance the positive and negative effects of astrocyte scar with traditional operations, such as STAT3-CKO in astrocytes (Anderson et al., 2016). Enhancing the sealing function or promoting astrocyte scar formation can reduce the damaged area and provide better protection to intact tissues, whereas the later regeneration of axons in the damaged core will become difficult (Faulkner et al., 2004; Anderson et al., 2016). Preventing scar formation will cause the diffusion of the damage-associated molecular patterns and enlarge the area of the damaged core (Faulkner et al., 2004; Bellver-Landete et al., 2019; Fu et al., 2020) and is not conducive to tissue remodeling and functional reconstruction. For chronic scars, existing treatment methods produce secondary damage and cause further astrocyte activation, such as a dissolved extracellular matrix (ECM) in the damaged area by chondroitinase ABC (Bradbury et al., 2002; Rao et al., 2018; Lien et al., 2019; Rosenzweig et al., 2019). Therefore, there is an urgent need to develop a method that can reduce the inhibition of astrocyte scar to an axon without compromising its sealing effect.

Recent advances have found that microglia are necessary for the activation of astrocytes in response to SCI to form the compact astrocyte scar sealing injury regions and that deficiency of microglia leads to a loose arrangement of astrocytes in the injury regions (Bellver-Landete et al., 2019). In addition, studies on scar formation mechanisms suggest that astrocytes involved in scar formation are predominantly present in scar-forming states. However, they can revert to a naive state if induced by the surrounding environment (Hara et al., 2017). This evidence suggests that we could alter the state of astrocyte scars by modulating microglia to achieve appropriate scar levels and improve axonal regeneration.

In this study, we sustained the microglia-deficient state by administering the colony-stimulating factor 1 receptor (CSF1R) inhibitor pexidartinib (PLX3397) to mice. Microglia depletion prevented the formation of compact astrocyte scars without increasing the damaged core area and promoted axon regeneration. We also observed that complete and stable microglia deletion in the chronic stage of injury could loosen existing compact astrocyte scars and, similarly, did not cause a marked expansion in the injury area. The loose astrocyte scars were correlated with decreased expression and an altered distribution pattern of microglia-derived collagen I in the damaged core region.

## Materials and Methods

### Animals

C57BL/6N mice (aged 8–10 weeks) were obtained from the Beijing Vital River Laboratory Animal Technology Co., Ltd. and were housed at the Laboratory Animal Center of the Institute of Zoology under standard conditions: 12/12 h light/dark cycle, free access to food and water, noise below 60 dB, a constant comfortable temperature (20–26°C), and humidity (40–70%) Supplementary Video. We ensured that animals did not experience unnecessary pain or fear. All animal experiments were approved by the Animal Care and Use Committees of the Institute of Zoology, Chinese Academy of Sciences (IOZ-IACUC-2021-105).

### Microglia Deletion

To delete microglia in mice, PLX3397 (Selleckchem), a CSF1R inhibitor, was formulated into AIN-76A standard diet at 290 and 600 mg/kg. In order to pre-delete microglia, mice were fed with medicated feed for 21 days (or mentioned in the article) before receiving an SCI-inducing surgery. To delete microglia in mature scars, the medicated feed was administered 10 weeks after the surgery (when scar formation was complete and remained stable) and continued for 10 weeks. An AIN-76A standard diet was used as the corresponding vehicle.

### SCI Surgery

The procedure of C57BL/6N mouse T9 spinal cord crush is similar to what was described previously (Li et al., 2020) with modifications. This type of injury is similar to the SCI caused by spinal fracture or dislocation, which is clinically common (Chiu et al., 2017; Bradbury and Burnside, 2019). Briefly, mice were anesthetized via an intraperitoneal injection of pentobarbital (75 mg/kg). A longitudinal incision was made on the shaved backs of the mice to cut open the skin and muscles to expose the thoracic vertebrae. Next, laminectomy was performed at T9 to expose the spinal cord, and the ninth thoracic level was crushed using forceps (JZ, WA3010) with a tip width less than 1 mm. The tip was closed for 10 s. The dura was kept intact throughout the injury process to avoid invasion of the spinal cord by muscle tissues during subsequent healing. Laminectomy was performed in the sham group, but without the crush-induced injury. Next, the open muscles and skins were sutured. Finally, mice were placed at 37°C to recover from anesthesia until their thermoregulation was re-established. Urine was emptied by manual abdominal pressure daily throughout the experiment.

### Immunofluorescence

Mice were perfused with normal saline under deep anesthesia to replace blood, followed by 4% paraformaldehyde (PFA) for 10 min. The 20 mm spinal cord segments, including the injured portion, were excised and placed in 4% PFA overnight and in 30% sucrose solution for 2 days to dehydrate. Frozen and dehydrated segments were cut into 20 μm thick sections and blocked with 0.5% Triton-X 100 and 5% bovine serum albumin for 1 h. The samples were incubated with primary antibodies [rat anti-glial fibrillary acidic protein (GFAP), 1:400, Invitrogen, 13-0300; rabbit anti-GFAP, 1:400, Abcam, ab7260; mouse anti- GFAP, 1:500, Millipore, MAB360; rabbit anti-β-Tubulin III (Tuj1), 1:1,000, Sigma, T2200; mouse anti-Tuj1, 1:1,000, T8660; mouse anti-Tuj1, 1:1,000, BioLegend, 801201; goat anti-Iba1, 1: 400, Abcam, ab5076; mouse anti-Iba1, 1: 400, Abcam, ab15690; rabbit anti-collagen I, 1: 400, Abcam, Ab21286] at 4°C overnight. After rinsing with phosphate-buffered saline (3 rinses, 5 min each), sections were incubated with secondary antibodies (Alexa Fluor^®^ 488-labeled donkey anti-rabbit IgG antibody, 1:1,000, Thermo Fisher, Waltham, MA, United States; Alexa Fluor^®^ 568-labeled donkey anti-mouse IgG, 1:1,000, Thermo Fisher; Alexa Fluor^®^ 647-labeled donkey anti-goat IgG antibody, 1:1,000, Thermo Fisher) at 25°C for 2 h in the dark. Finally, sections were rinsed with phosphate-buffered saline and mounted with a DAPI-containing sealant. The samples were observed under a laser confocal microscope (880 Ariyscan, Leica, Wetzlar, Germany). No primary antibodies were used in the controls.

### Western Blotting

Spinal cord segments (2 mm long, including the injured portion) were homogenized in an ice-cold RIPA Lysis and Extraction Buffer (89901; Thermo, CA, United States) supplemented with a protease inhibitor cocktail (78439; Thermo, CA, United States). The total protein concentration was determined by the bicinchoninic acid method (23250; Thermos, CA, United States) and was separated by sodium dodecyl sulfate-polyacrylamide gel electrophoresis at 100 V for 2 h. Separated proteins were electro-transferred onto polyvinylidene difluoride membranes at 300 mA for 90 min. The membranes were blocked with Tris-buffered saline with Tween 20 (TBST) containing 5% skimmed milk powder for 2 h and incubated with the primary antibodies (rabbit monoclonal anti-collagen I, 1:250, Abcam, ab21286; mouse monoclonal anti-GAPDH, 1:4,000; AF0006, Beyotime) at 4°C overnight. After rinsing with TBST (three rinses, 15 min each), the membranes were incubated with secondary antibodies (horseradish peroxidase-conjugated goat anti-rabbit or anti-mouse IgG, 1:1,000, Abcam) at room temperature for 2 h. Immunoreactive bands were visualized with an enhanced chemiluminescence reagent (ECL, Pierce) and quantified using ImageJ software (version 1.53c).

### Fluorescence Labeling of Axons

The virus AAV 2/9 rAAV-hSyn-EGFP-WPRE-SV40 polyA (Titer: 2.71E+12 ug/ml Cat# PT-0905, BrainVTA) was used to label the descending spinal tract. A stereotaxis apparatus was placed on both hemispheres of mice brains to construct the coordinates, with the bregma being the coordinate origin. 1 mm beneath the meninges, 0.5 μL of the virus was injected at six selected points, (1.5, 0), (3.0, 0), (1.5, 1.5), (−1.5, 0), (−3.0, 0), and (−1.5, 1.5). Samples were collected 2 weeks after the virus injection surgery.

### Behavioral Analyses

The hindlimb motor function in mice was evaluated with the locomotor open-field Basso Mouse Scale (BMS), following the method developed by Basso et al. (Basso et al., 2006). Behavioral records were taken at the same time every week since the date of surgery. Two experienced examiners evaluated each mouse for 4 min and assigned an operationally defined score for each hindlimb.

### Statistical Analysis

The statistical significance of data was assessed by Student’s two-tailed paired/unpaired *t*-test between two groups and one-way ANOVA between three groups using Prism v8.0 (GraphPad Software, Inc.). Data distribution was assumed to be normal. Data are presented as the mean ± standard error of the mean. The statistical significance (P) was set at 0.05.

## Results

### Microglia Deletion Induced Incompact Astrocyte Scarring but Retained its Sealing Ability

**FIGURE 1:**
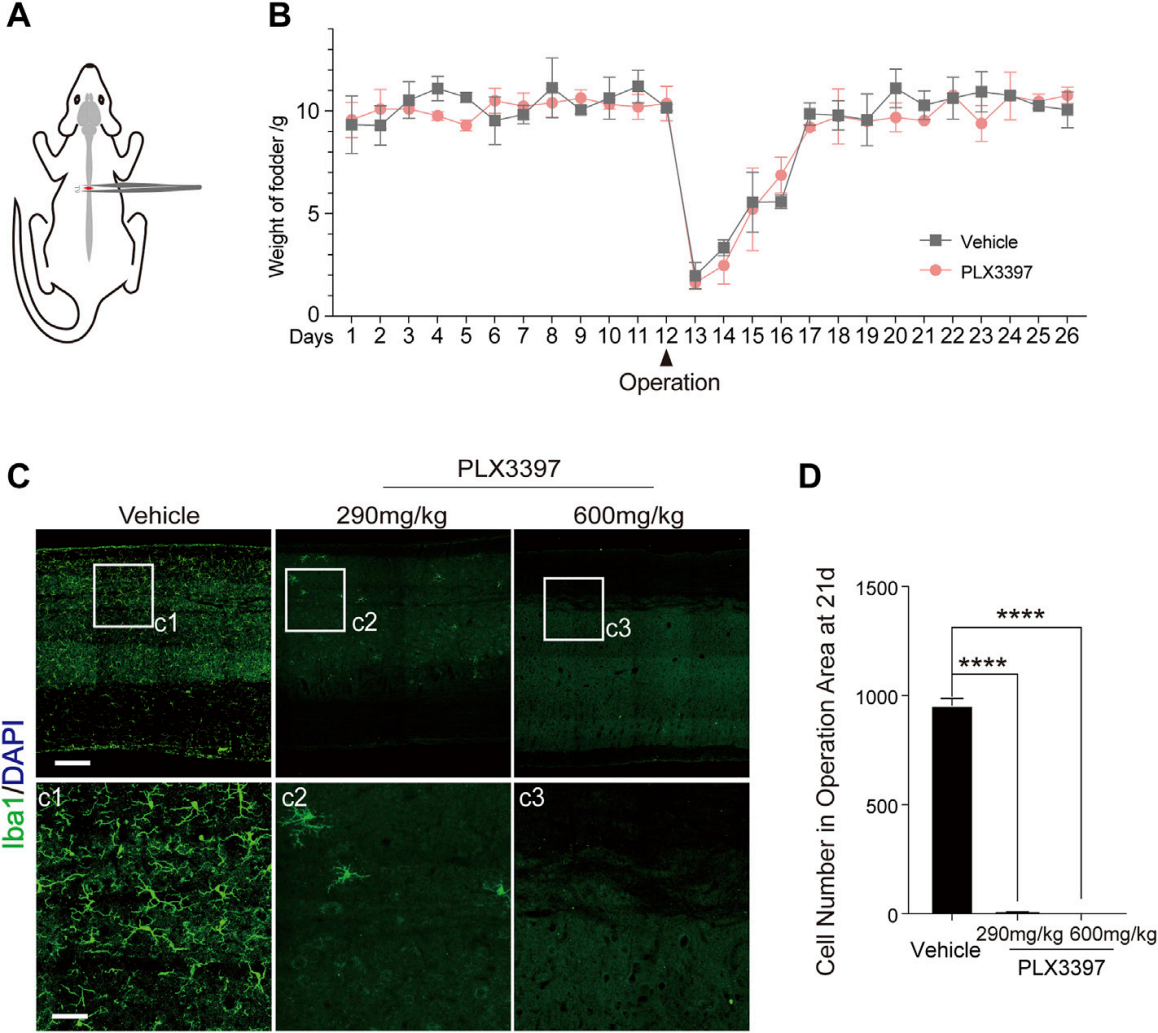
SCI model and microglia deletion strategy validation. **(A)** Schematic diagram of the surgical procedure and injury location in the SCI model. **(B)** Changes in animal food intake before and after SCI surgery (each datum represents the 24 h feeding weight of five mice, with three replicates in each group, aged 6–10 weeks) **(C)**. Immunofluorescence staining was used to detect the deletion of microglia (Iba1, green) under different treatment conditions (vehicle diet, PLX3397-290 mg/kg for 21 days, PLX3397-600 mg/kg for 21 days), scale bar = 500 μm; c1-c3 shows enlarged microglia, scale bar = 125 μm. **(D)** Microglia elimination efficiency statistics (*n* = three repeats, mean ± SD, ****p < 0.0001, significant difference among means, ordinary one-way ANOVA).

To detect the effect of microglia depletion on astrocyte scar after SCI, we adopted the T9 crush injury of the spinal cord in C57BL/6N mice (Figure 1A, Supplementary Figure S1A). To delete microglia, we fed the mice with PLX3397, a CSF1R inhibitor. This approach can specifically delete microglia from the central nervous system without significantly affecting astrocytes (Supplementary Figure S2A) (Elmore et al., 2014; Ma et al., 2020). We observed that the subject mice had a sharp decrease in food intake within 4 days after SCI surgery (Figure 1B and Supplementary Figures S1B,C; a decrease by over 80% on the first day after surgery and over 50% on the first 4 days), which may have resulted in insufficient PLX3397 intake. Meanwhile, when CSF1R inhibition was removed, microglia in subject mice repopulated at ∼20% per day (Huang et al., 2018; Ma et al., 2020). These may have together caused subsequent incomplete microglia deletion. Considering the potential effects of gavage on mice after non-healing wounds and damaged vertebrae (Bellver-Landete et al., 2019), a PLX3397 concentration of 600 mg/kg was used to ensure minimal participation of microglia in astrocyte scar formation (Elmore et al., 2014) (Figures 1C,D).

**FIGURE 2:**
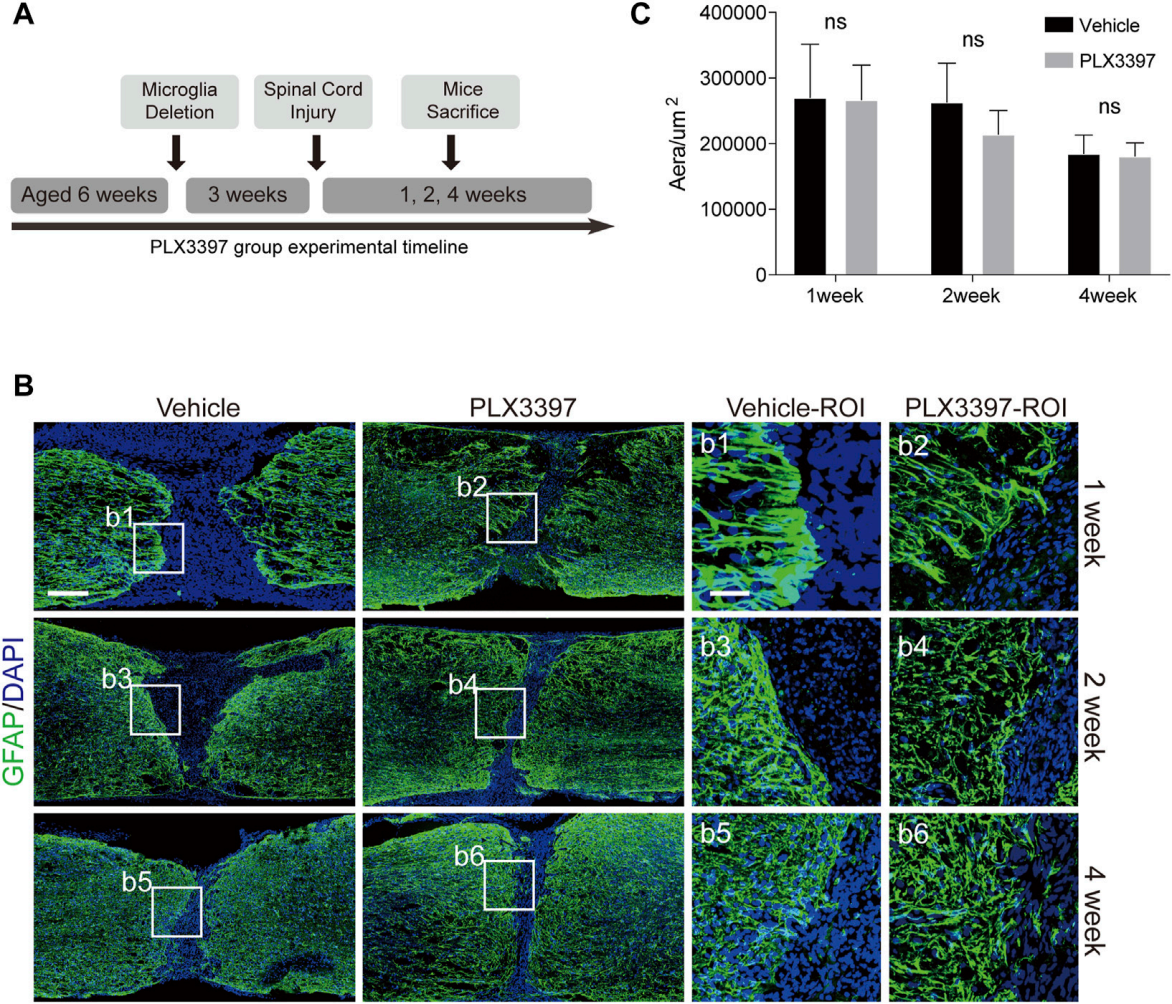
Deletion of microglia induced reactive astrocytes to be distributed loosely at scar boundaries. **(A)**. Experimental timeline for microglia deletion and SCI surgery in C57BL/6N mice. “Microglia Deletion” indicates the time to start administering the PLX3397 feed. **(B)**. Immunofluorescence staining was used to detect astrocyte scars (GFAP in green) at different time points (1, 2, and 4 weeks). The nucleus is marked with DAPI in blue, scale bar = 500 μm; b1-b6 shows an enlarged image of the area of interest, scale bar = 125 μm. **(C)**. Area of the damaged core (GFAP negative area of the injured area) was statistically compared (*n* = three repeats, mean ± SD, *p* = 0.3355, no significant difference, paired *t*-test).

Microglia were deleted by administering PLX3397 at 600 mg/kg 3 weeks prior to the SCI surgery (Figure 2A). Astrocyte scar formation defects were observed in the microglia deletion group (Figure 2B), and astrocytes were present in the non-scar-forming state [a low activation state, Figure 2B(b1-b6)].

To detect whether the disruption of astrocyte scar during the operation caused the spread of the damaged core area, we compared the damaged core area of the two groups and found no significant expansion of the injury area when microglia were deleted (Figure 2C). Therefore, the loose astrocyte scar caused by the deletion of microglia retained its ability to isolate damage factors.

### Microglia Deletion-Induced Incompact Astrocyte Scarring Increases the Axon Density in the Damaged Core

**FIGURE 3:**
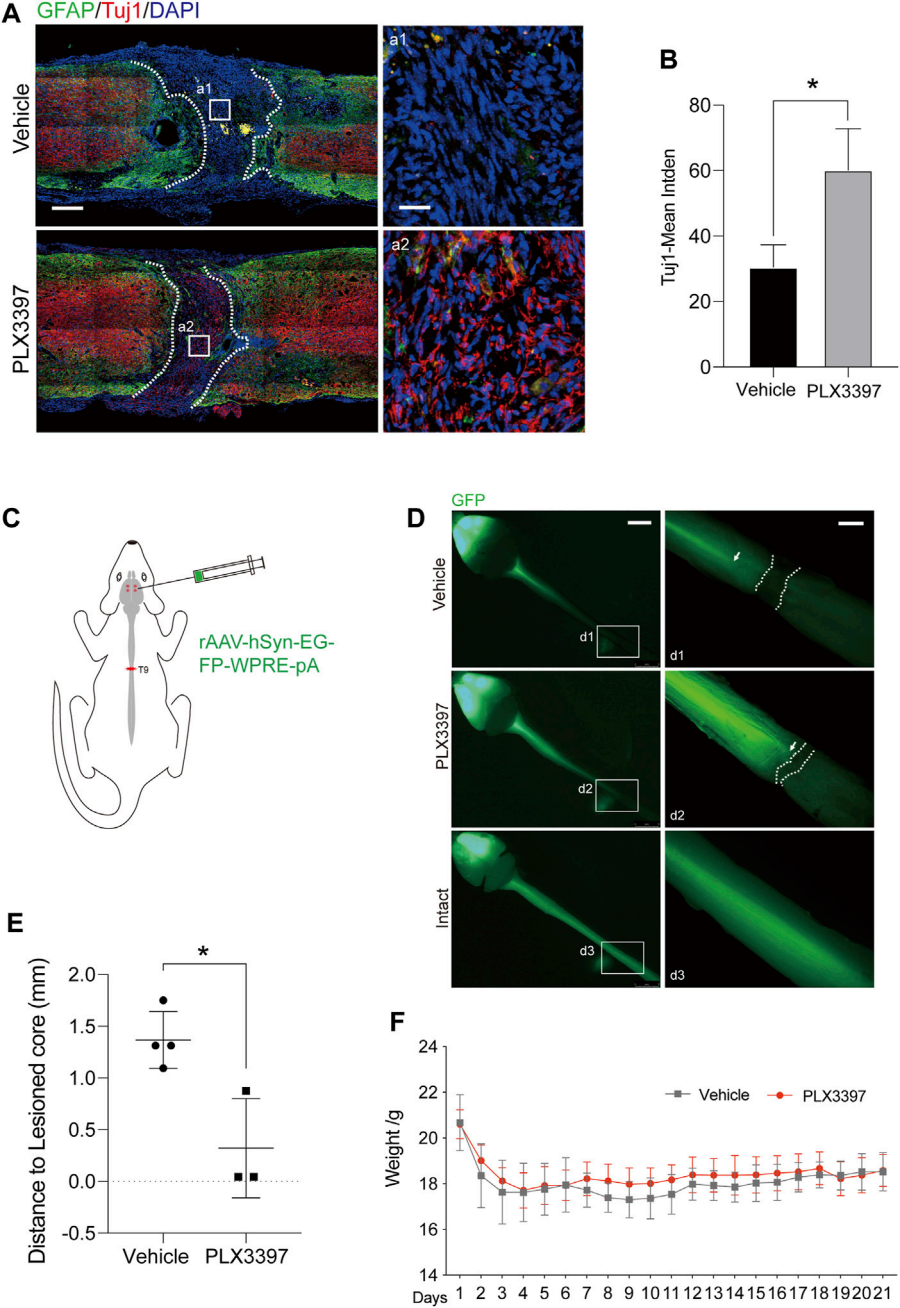
Deletion of microglia promotes the growth of axons in the damaged core. **(A)**. Immunofluorescence staining was used to distinguish the different area and indicates the extent of the axon, 10 weeks after surgery-induced astrocyte scar (GFAP, green) and the axon (Tuj1, red), scale bar = 500 μm; a1-a2 shows the magnified area of interest, scale bar = 50 μm. **(B)**. Statistics of Tuj1-mean integrated density in different groups (n = three repeats, mean ± SD, *p = 0.0233, significant difference at *p* < 0.05, unpaired *t*-test) **(C)**. Schematic diagram of trace virus injection (AAV 2/9 rAAV-hSyn-EGFP-WPRE-SV40 polyA) **(D)**. Fluorescence image of the central nervous system of C57BL/6N mice, where the green fluorescence (eGFP) shows the corticospinal tract projected from the cerebral cortex to the spinal cord, scale bar = 5 mm; d1-d3 shows the enlarged images of the area of interest, scale bar = 1 mm. **(E)**. Statistics of distance from termination of the spinal cord fluorescence signal (arrows) to the scar boundary (dotted line) (n ≥ three repeats, mean ± SD, *p = 0.0141, significant difference at *p* < 0.05, unpaired *t*-test). **(F)**. Weight changes in experimental animals after surgery. n = 10 repeats, mean ± SD, days 8–10, *p < 0.05, significant difference among means, paired *t*-test.

The most harmful effects of compact astrocyte scar on the central nervous system are the deletions of neurons and axons in injured core areas and inhibition of axon regeneration (Liddelow et al., 2017; Sofroniew and Vinters, 2010; Chung et al., 2013). To observe the effect of loose astrocyte scar on axon regeneration, we detected the distribution of axons in the damaged area of the loose scar group (PLX3397 group) mice at 10 weeks after SCI. The results showed that the distribution range of neurons in the loose astrocyte scar group was closer to the astrocyte scar boundary (Figure 3A). At the same time, in the PLX3397 group, the damaged spinal tract had a higher density of axons than the vehicle group in the injury core (Figures 3A,B). A similar result was observed when using a fluorescent virus to track the spinal tract’s downward projection: axons were present closer to the scar boundary in the loose group than in the vehicle group (Figures 3C–E). The weight of the mice was recorded as an index to evaluate the overall health status (Sulzbacher et al., 2022). We found that the body weight of the PLX3397 group was slightly higher than that of the wild type after surgery (Figure 3F and Supplementary Figure S1D). These results showed that the health status of the PLX3397 group was better than that of the wild type. These results suggested that the incompact astrocyte scar induced by microglia deletion had a positive effect on axon regeneration and the health status of mice.

### Decreased Collagen I Expression Induced the Loosening of Astrocyte Scar

**FIGURE 4:**
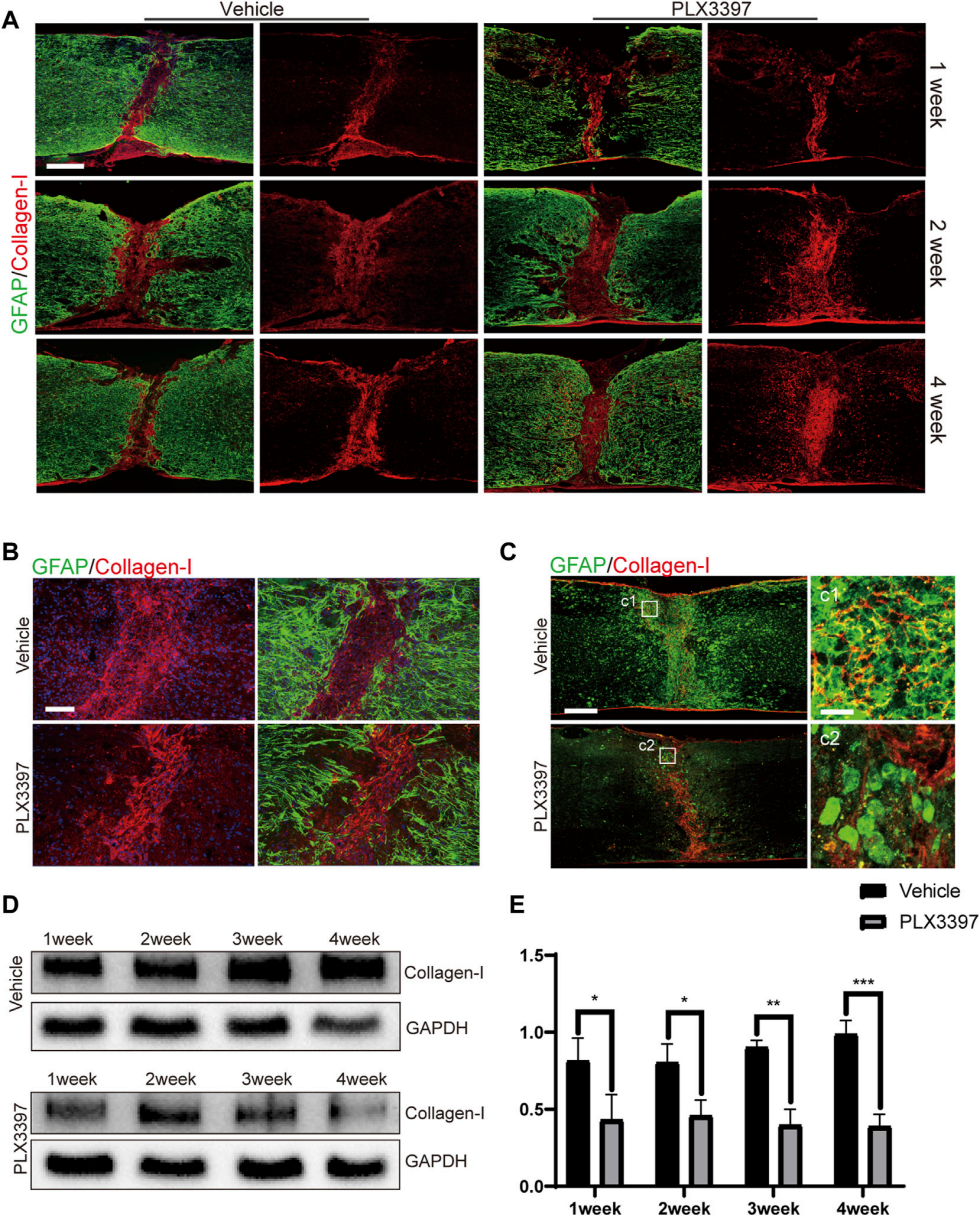
Reduced collagen I expression induced incompact astrocyte scar formation. **(A)**. Representative confocal images for astrocyte scar (GFAP, green) and collagen I (collagen I, red) at different time points (1, 2, and 4 weeks post SCI) show collagen I merged with GFAP and separate collagen I. Scale bar = 500 μm. **(B)**. Representative confocal images of the astrocyte scar border (GFAP, green) and collagen I (collagen I, red) core (post operation 1 week) show collagen I separately and collagen I merged with GFAP. Scale bar = 100 μm. **(C)**. Representative confocal images for collagen I (red) co-localization with microglia/macrophages (Iba1, green), scale bar = 500 μm; d1-d2 shows the magnified area of interest, scale bar = 30 μm. **(D)**. Representative western blots of collagen I expression levels in the injured part of the spinal cord from each group. **(E)**. Quantitative analysis of collagen I expression; the integrated density ratio of collagen I and GAPDH was taken as the ordinate (*n* = 3 repeats, mean ± SD, *p < 0.05).

Astrocyte activation requires the participation of ECM (Ridet et al., 1997; Hara et al., 2017) and multiple cytokines (Liddelow et al., 2017; Schiweck et al., 2021). Collagen I is an ECM secreted by multiple cells within a few days after injury (Shintani et al., 2006; Yonezawa et al., 2010; Dias et al., 2018) and is required for astrocyte activation. Additionally, we observed that microglia are an important source of collagen I (Jones et al., 2002; Li et al., 2020). Therefore, we speculated that microglia deletion would lead to reduced collagen I expression and thus prevent astrocytes from forming compact astrocyte scars. Immunofluorescence staining showed that collagen I remained concentrated in the damaged core area at all time points in the vehicle group (Figure 4A) and showed a strong co-localization association with microglia/macrophages (as activated microglia were difficult to distinguish from macrophages in morphology) (Figure 4C). In contrast, collagen I in the loose astrocyte scar group dispersed 1 week post injury (Figures 4A,B), and further observation detected no co-localization between collagen I and microglia/macrophages in the damaged core area (Figure 4C). Furthermore, we measured collagen I expression in loose astrocyte scar using western blotting and found a downregulated collagen I expression in the subacute phase (1–4 weeks) of SCI, in response to microglia deletion (Figures 4D,E). These results indicated that microglia deletion would decrease collagen I expression and change the distribution pattern in the damaged area such that activated astrocytes would exit the scar-forming state and no longer generate compact scars.

### Microglia Deletion Disrupted the Compact Arrangement of Chronic Astrocyte Scars

**FIGURE 5:**
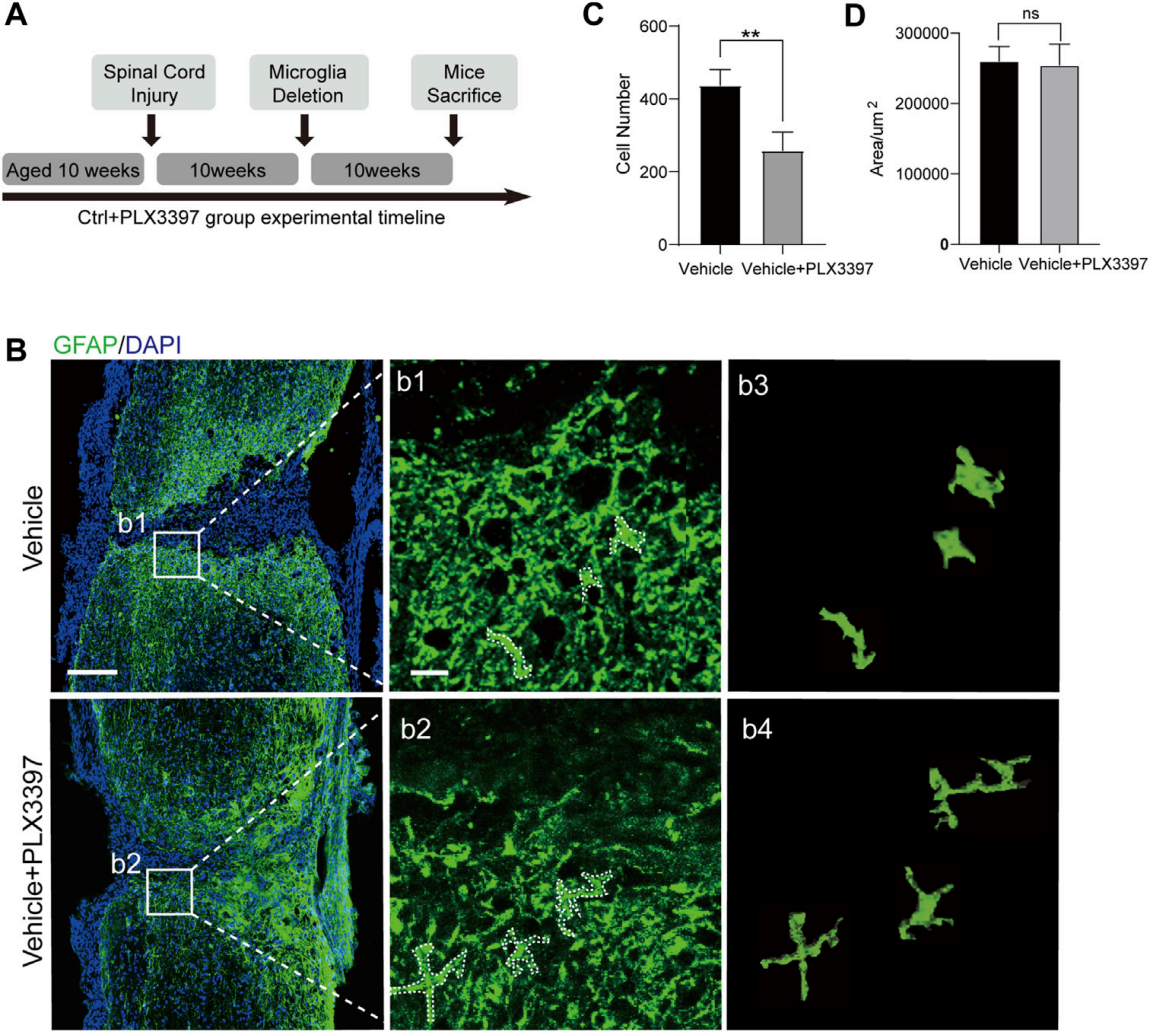
Deletion of microglia attenuated the activation of chronic astrocyte scar. **(A)** Experimental timeline for SCI surgery in C57BL/6N mice and the procedure of deleting microglia in chronic astrocyte scars. “Microglia Deletion” indicates the time when the PLX3397 feed was started. **(B)** Representative confocal images for chronic astrocyte scar changes caused by microglia deletion (GFAP, green), scale bar = 500 μm; b1-b2 shows the magnified area of interest, scale bar = 50 μm; b3 shows the morphology of the cells selected in the dotted box of b1; b4 shows the morphology of the cells selected in the dotted box of b2. **(C)** Statistics of astrocyte (GFAP, green) quantity in the astrocyte scar border; the cell counting region was astrocyte scar, and a 2.5 × 10^5^ μm^2^ region was taken from the center and edge of the spinal cord, *n* = 3 repeats, means ± SD, *p = 0.0097, significant different at *p* < 0.05, unpaired *t*-test. **(D)** Area of the damaged core (GFAP negative area of the injured area) was statistically compared (*n* = 3 repeats, mean ± SD, *p* = 0.7999, no significant difference, paired *t*-test).

In the absence of microglia, astrocyte scars will not completely form in the pathological process of SCI, leading to an unclear scar boundary (Bellver-Landete et al., 2019). However, it is not clear whether microglia are required to maintain mature astrocyte scars. To verify that the same, we deleted microglia in mice 10 weeks (Parvin et al., 2021) post SCI and collected their spinal cord samples after another 10 weeks (Figure 5A). The results showed that the deletion of microglia in chronic scars led to the collapse of the compact scar boundaries (Figure 5B). Through further observation, we found a morphological restoration of astrocytes from the scar-forming state (having reduced dendrites and hypertrophy of the cell body) to the non-scar-forming state (having increased dendrites and a thin cell body, Figure 5B, b1-b4). Cell counting showed that the number of astrocytes in astrocyte scar decreased after deletion of microglia, whereas the area of scar did not increase (Figures 5C,D). In summary, microglia deletion in the chronic stage of astrocyte scars disrupted the compact scar arrangement by inactivating astrocytes. Therefore, microglia were required for both the formation and maintenance of compact astrocyte scars.

## Discussion

Astrocyte scar, as an important pathological structure after SCI (Bradbury and Burnside, 2019), has been attracting continuous attention from researchers, and its role has been widely debated (Liddelow and Barres, 2016; Silver, 2016; Liddelow and Barres, 2017; Escartin et al., 2021). The gaps between the incisions will continue to exist and cannot be recaptured by glial cells or neurons and may eventually develop into a series of cystic cavities filled with the tissue fluid (Bradbury and Burnside, 2019). There is consensus that this pathological structure isolates the damaged area and hinders axon regeneration (Anderson et al., 2018). In our view, it is important to understand whether the regenerative barrier can be removed without breaking the isolation. Traditional astrocyte scar treatment methods are often complex (Bradbury et al., 2002; Ropper et al., 2017; Anderson et al., 2018; Rosenzweig et al., 2019; Ceto et al., 2020), and the side effects should be examined before considering the sealing damage. The preservation of scars appears to be a trade-off (Anderson et al., 2016). Recent studies have shown that the reactivity of astrocytes is affected by the local microenvironment (Hara et al., 2017; Dias et al., 2018; Rothhammer et al., 2018; Bellver-Landete et al., 2019; Joshi et al., 2019). For example, astrocytes can be in either a naive state or a scar-forming state, which are reversible and are related to collagen I (Hara et al., 2017). Our study further showed that the activated microglia in response to SCI were an important source of collagen I. More importantly, decreased collagen I expression and changes in the distribution pattern caused by the deletion of microglia could relieve the inhibitory effect of astrocyte scars on axon regeneration without significantly compromising the isolation effect. This discovery is helpful to further understand the formation mechanisms of astrocyte scars. As an astrocyte scar intervention, there is high potential for clinical applications of deleting microglia.

Microglia perform immune functions and participate in inflammatory responses after SCI (Wang et al., 2019; Ma et al., 2020; Favuzzi et al., 2021). The interactions between astrocytes and microglia have recently been elucidated (Liddelow et al., 2017; Bellver-Landete et al., 2019; Hutchinson and Isaacson, 2019). For example, microglia in the inflammatory environment can induce reactive astrocytes through IL-1α, TNF, and C1q. Reactive astrocytes (termed in the A1 state) then kill neurons in the damaged area (Liddelow et al., 2017). Microglia are also essential for astrocytes to form astrocyte scars after SCI. Similar to our results, loose scar boundaries have been observed after deletion of microglia (Bellver-Landete et al., 2019). However, previous studies observed an expansion in the damaged area after the loosening of the boundary, which may be influenced by various reasons. We speculate that the manner in which microglia are deleted could be a possible reason for the same. Compared to the previous studies, we deleted microglia more thoroughly by adopting the high-concentration CSF1R inhibitor PLX3397. Another similar phenotype was the stronger regeneration after microglia deletion as previous data showed that the larger original damaged area in the microglia deletion group became smaller and comparable to that of the vehicle group after a few weeks.

Unexpectedly, we observed stronger axon regeneration after microglia deletion in the experimental group when compared to that in the vehicle group, with no significant behavioral differences (Supplementary Figure S1E, Supplementary Figures S2B,C). We noticed that in the BMS scoring system, all our subject mice had BMS scores lower than 4, even over long periods. Two possible reasons could be that the injury to the model was so severe that even the enhanced axon regeneration was not enough to improve the behavioral score and the dormancy of neurons after SCI prevented the structural reconstruction from being reflected as a functional reconstruction (Chen et al., 2018; Brommer et al., 2021). This needs to be further explored in subsequent studies.

Overall, we subjected mice to SCI and microglia deletion. We observed decreased expression and altered distribution of collagen I in the damaged area in response to microglia deletion, which further induced astrocytes to exit the scar-forming state. Compact astrocyte scars then loosened but retained their ability to seal the damaged area from expanding. In addition, the loose astrocyte scars allowed enhanced axon regeneration. Our study helps to further understand the formation mechanisms of astrocyte scars after injury to the central nervous system, and microglia deletion by a CSF1R inhibitor may help improve functional recovery and structural reconstruction in the damaged area after SCI treatment.

## Data Availability

The original contributions presented in the study are included in the article/Supplementary Material; further inquiries can be directed to the corresponding authors.
