# Supplementary figures and images for: Continual Deletion of Spinal Microglia Reforms Astrocyte Scar Favoring Axonal Regeneration
Source: Front Pharmacol. 2022 Jun 27;13:881195. doi: 10.3389/fphar.2022.881195 (PMC9271995; doi:10.3389/fphar.2022.881195)

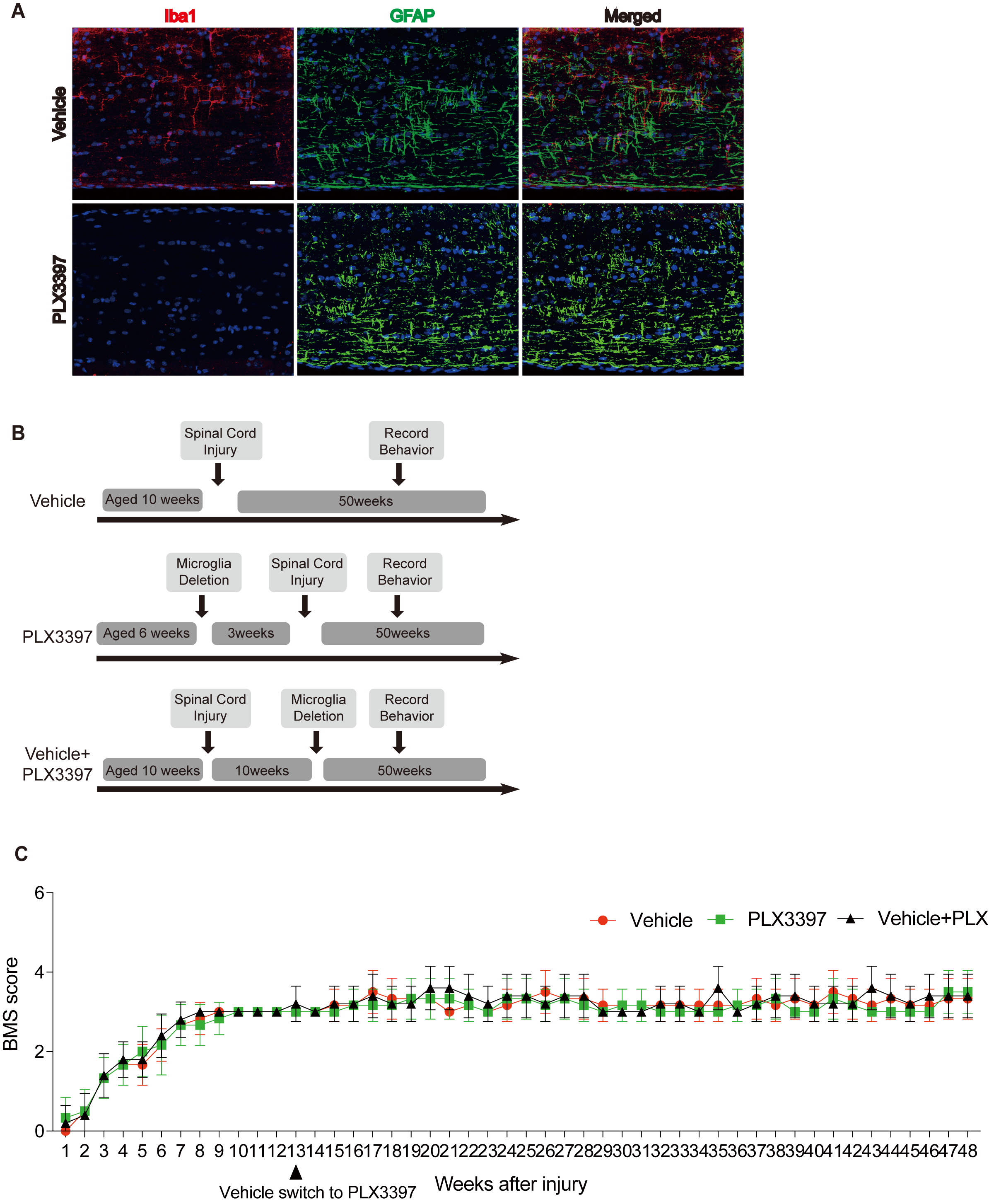

Supplement: Supplementary file 1 [file Image2.TIF]

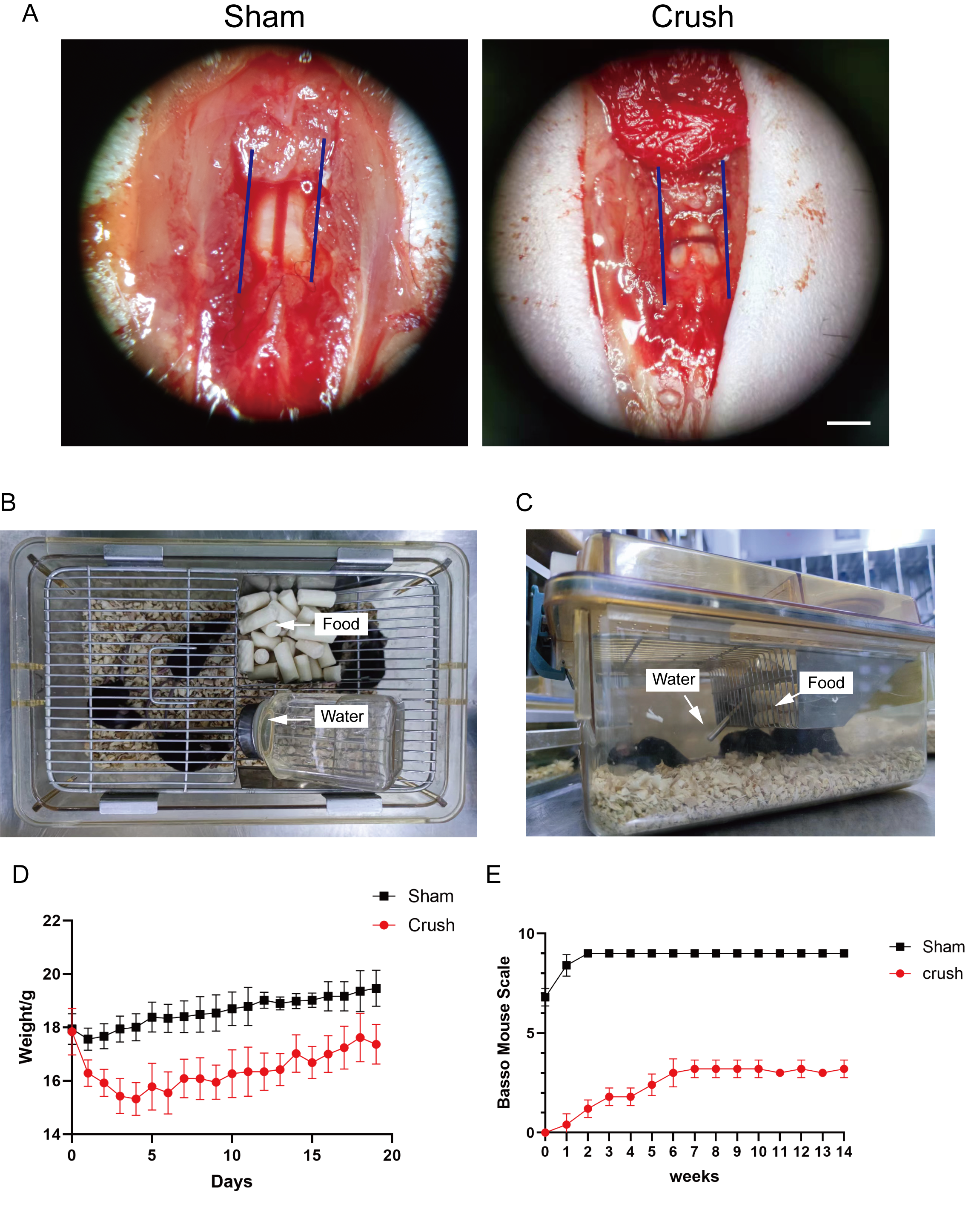

Supplement: Supplementary file 2 [file Image1.TIF]
